# Development of Synchronized High-Sensitivity Wireless Accelerometer for Structural Health Monitoring

**DOI:** 10.3390/s20154169

**Published:** 2020-07-27

**Authors:** Shaik Althaf Veluthedath Shajihan, Raymond Chow, Kirill Mechitov, Yuguang Fu, Tu Hoang, Billie F. Spencer

**Affiliations:** 1 Department of Civil and Environmental Engineering, University of Illinois at Urbana-Champaign, Urbana, IL 61801, USA; sav4@illinois.edu (S.A.V.S.); tuhoang2@illinois.edu (T.H.); 2 Epson America Inc., San Jose, CA 95112, USA; Raymond.Chow@ea.epson.com; 3 Department of Computer Science, University of Illinois at Urbana-Champaign, Urbana, IL 61801, USA; mechitov@illinois.edu; 4 School of Mechanical Engineering, Purdue University, West Lafayette, IN 47907, USA; fu303@purdue.edu; 5 Anne M. and Nathan M. Endowed Chair in Civil Engineering, University of Illinois at Urbana-Champaign, Urbana, IL 61801, USA

**Keywords:** synchronized sensing, high-sensitivity accelerometer, wireless smart sensor, digital output sensor, structural health monitoring, time synchronization

## Abstract

The use of digital accelerometers featuring high sensitivity and low noise levels in wireless smart sensors (WSSs) is becoming increasingly common for structural health monitoring (SHM) applications. Improvements in the design of Micro Electro-Mechanical System (MEMS) based digital accelerometers allow for high resolution sensing required for SHM with low power consumption suitable for WSSs. However, new approaches are needed to synchronize data from these sensors. Data synchronization is essential in wireless smart sensor networks (WSSNs) for accurate condition assessment of structures and reduced false-positive indications of damage. Efforts to achieve synchronized data sampling from multiple WSS nodes with digital accelerometers have been lacking, primarily because these sensors feature an internal Analog to Digital Converter (ADC) to which the host platform has no direct access. The result is increased uncertainty in the ADC startup time and thus worse synchronization among sensors. In this study, a high-sensitivity digital accelerometer is integrated with a next-generation WSS platform, the Xnode. An adaptive iterative algorithm is used to characterize these delays without the need for a dedicated evaluation setup and hardware-level access to the ADC. Extensive tests are conducted to evaluate the performance of the accelerometer experimentally. Overall time-synchronization achieved is under 15 µs, demonstrating the efficacy of this approach for synchronization of critical SHM applications.

## 1. Introduction

Structural health monitoring (SHM) involves the assessment of civil infrastructure for operational safety, integrity, and remaining useful life estimation. Some of the recent tragic incidents involving newly constructed structures (e.g., the collapse of Xinjia Hotel in Quanzhou, China leaving 29 people dead [1], and the collapse of FIU pedestrian bridge in Miami, USA which left six people dead [2]) show the impending dangers existing even among modern-day infrastructure. These lessons, in addition to aging structures globally reaching their design life, necessitate the need for moving towards long-term periodic monitoring of structures rather than waiting for the onset of visible damage. Traditionally, sensor-based monitoring has been carried out using wired systems as an alternative to the intermittent visual inspection by engineers. Accelerometers are among the most commonly used sensors for SHM owing to their inherent nature to capture the reference-free dynamic characteristics of a structure. However, wired networks incur a high initial cost of instrumentation, require frequent maintenance, and scalability is limited due to cabling. Especially for large-scale structures such as long-span bridges, wired SHM systems can be prohibitively expensive, where the installed cost of each sensor channel can cost $5000 to $15,000 [3]. Over the past decade, a shift from the traditional wired monitoring systems to wireless sensing has taken place due to numerous benefits, such as lower costs, scalability, less invasive deployment, reusability, and real-time remote monitoring capabilities. Moreover, the arrival of low-cost and low-power MEMS-based inertial sensors have made the new-generation of wireless smart sensors (WSSs) with on-board processing capabilities a valuable asset for SHM applications.

Unlike event-based monitoring (such as for earthquakes and storms), where large vibrations induced on a structure is measured, long-term periodic monitoring is usually based on the low-level ambient vibration. Often, these vibration levels are so small that they cannot be perceived by humans; however, if measured using a sensor with sufficient resolution, valuable information about the dynamics of the structure can be collected for operational modal analysis (OMA). A detailed comparison of available low-power WSS accelerometers presented by Li et al. [4] shows that in the low-frequency range, the majority have a threshold of approximately 0.05 mg. A review by Ragam et al. [5] emphasizes the need for high-resolution accelerometers in WSSNs capable of measuring microvibration due to blast-induced ground vibration (BIGV) for monitoring and protecting surrounding structures. Wind turbine monitoring carried out by Wondra et al. [6] in Bavaria, Germany, showed that WSSs with accelerometer resolution in the order of 1 mg were insufficient to analyze the behavior of the structure during low wind speeds. A long-term monitoring study conducted by Darbre and Proulx [7] on a 250 m concrete arch dam in Mauvoisin, Switzerland, using wired high-sensitivity force-balanced accelerometers shows ambient-vibration to be on the order of a few µg’s, which cannot be measured accurately using WSS accelerometers currently on the market.

To meet the high-resolution demands of long-term periodic monitoring of infrastructure, Li et al. [4] proposed the integration of external high-sensitivity digital output accelerometers, having a resolution in the order of µg, with WSSs. However, as we move towards external digital output sensors, we are faced with a unique set of challenges concerning synchronized sensing. Muttillo et al. [8] proposed a wireless system for indoor monitoring using an ADXL355, which is a high-resolution digital accelerometer. However, the case study reports the use of only one sensor node directly connected to two accelerometers for OMA of a beam-element in a building; therefore, the study did not explore the data synchronization aspect. Valenti et al. [9] present a WSSN for building monitoring using the ADXL355; however, the study reports a relatively large time-synchronization error of 6 ms at the application layer. By comparison, Li et al. [10] achieve synchronization error below 50 µs using WSSs, although with analog accelerometers.

Time synchronization is a nontrivial task even in a standard WSSN for SHM and requires twofold consideration: (1) clock synchronization; (2) data synchronization. Each sensor node in the network possesses its own independent processor with a local clock which may not be in synchronization with the other nodes. Beacon packets carrying timing information are exchanged between nodes to correct the local clock drift and accommodate clock skew. Well-established communication protocols such as Flooding Time Synchronization Protocol (FTSP) [11] and Time-sync Protocol for Sensor Networks (TPSN) [12] are used for this task. However, they are designed to calibrate only the clock synchronization errors. Moreover, even clock synchronization in SHM applications faces some nontraditional challenges, which includes data acquisition at high-sampling rates, long-duration continuous sensing, nonlinear clock drifts arising from temperature variations among WSSs, and demand for rapid wake-up-response to sudden events. These challenges are discussed in detail for analog sensors in work by Li et al. [10]. Nevertheless, even perfectly synchronized clocks do not necessarily result in synchronized sensing.

Data synchronization on top of clock synchronization is used to achieve time-aligned data samples of the physical quantities being measured by the external sensors on multiple WSS nodes in a network. Nagayama and Spencer [13] categorized the primary sources of error in this task into three aspects: (1) random variation in sensing startup time among WSSs due to warm-up time and overhead in middleware services; (2) significant deviation from nominal clock frequency among nodes due to low-precision clock crystals used in low-cost sensors; and (3) sampling frequency fluctuation over time due to jitter in WSSs. The authors proposed an efficient resampling based approach using global time stamps to deal with these challenges. Implementation using the Imote2 [14] WSS on a laboratory truss-structure reported approximately 30 µs synchronization error for short-duration sensing. Over the past decade, the aforementioned sources of synchronization errors and different workaround strategies have been widely investigated [15,16,17,18,19,20,21]; thereby, these will not be dealt with in detail in this paper. Fu [22] discusses various time synchronization strategies and associated performance levels (clock and data synchronization errors) achieved by different researchers in WSSNs for SHM applications.

Synchronized data acquisition is crucial to accurate condition assessment and in reducing false alarms in damage detection of structures. It is not sufficient to have high-resolution capabilities for an individual sensor node, which can accurately measure the vibration amplitude at a particular location; the phase information gathered by the sensors distributed across the structure are just as important indicators of structural performance. Synchronization error of 1 ms in data can show up as 3.6 degree phase lag for a mode at 10 Hz, while for a higher mode at 100 Hz, it leads to a 36 degree phase shift [13]. Studies by Nagayama et al. [23] on the long-span suspension bridge indicate even a 3.6-degree phase shift in mode shape could lead to an erroneous conclusion. For damage detection using modal analysis, higher modes are known to be better indicators of structural damage; however, they are more sensitive to time synchronization errors—experiments conducted by Krishnamurthy et al. [24] show that even a time shift of 30 µs can result in a false-positive indication of damage at a location from the reconstruction errors at higher modes.

External digital accelerometers typically feature an internal microprocessor and clock crystal. In addition, in particular, they often come with an internal Analog to Digital Converter (ADC) to which the host device has no direct access, leading to additional uncertainty in the ADC startup time. Thereby, the actual timestamp corresponding to the start of sensing is not necessarily known with the level of certainty required for some of the SHM applications. Previous research on data synchronization has not addressed this issue with digital accelerometers for WSSs. In an extensive study conducted by Narayanan [25] for machine condition monitoring applications using a digital accelerometer (ADXL355 [26]), a data synchronization error of less than 50 µs was achieved. However, the selected accelerometer in the study had a feature which provided access to the accurate ADC timestamps, by allowing the changing of the master clock for the sensor to an external user-controlled clock along with an external-sync trigger pin. To minimize power consumption, features enabling access to the internal timestamp are not commonly available among different digital accelerometers in the market. Studies employing digital accelerometers with WSSs that have reported the achieved level of time synchronization error are summarized in Table 1.

In this paper, a high-sensitivity and low-power digital accelerometer featuring amongst the highest-resolution levels in this class of commercial accelerometers, the M-A352 (Seiko Epson Corporation, San Jose, CA, USA), is integrated with a next-generation WSS platform, the Xnode. Subsequently, the developed hardware and software framework to support long-term periodic monitoring of structures is presented. We propose a method to characterize and compensate for the ADC startup delay uncertainties observed in external digital accelerometers with WSSs using an adaptive iterative algorithm without the need for a dedicated evaluation setup and hardware-level access to the ADC. This approach can be generalized and extended to many of the available digital sensors. Thereby, we present a synchronized sensing framework for WSSNs, incorporating external digital accelerometers. Finally, experimental validation is carried out to evaluate the performance of the developed WSS for long-term condition monitoring and synchronized sensing.

## 2. Synchronized Sensing Framework for High-Sensitivity Accelerometer

We design a software framework and a hardware interface required to achieve synchronized data acquisition with an external digital output sensor using a next-generation wireless smart sensor platform, the Xnode. This section briefly overviews the Xnode architecture and summarizes the key features of the high-sensitive digital output accelerometer used in this study, Epson M-A352, which makes it a suitable candidate for SHM applications. In addition, it highlights the time synchronization strategies in SHM and presents the proposed synchronized sensing framework for digital output sensors.

### 2.1. Xnode

The Xnode WSS platform has undergone development in stages to meet the growing demands of full-scale SHM deployments and taking account of the lessons learned along the way [29,30,31,32,33]. Notably, to date, the Xnode features the world’s largest number of WSS nodes deployed for SHM of an infrastructure, where 192 Xnode’s are being used for monitoring the cable tension on the world’s tallest Ferris wheel, Ain Dubai in the United Arab Emirates [34,35]. 

**Figure 1:**
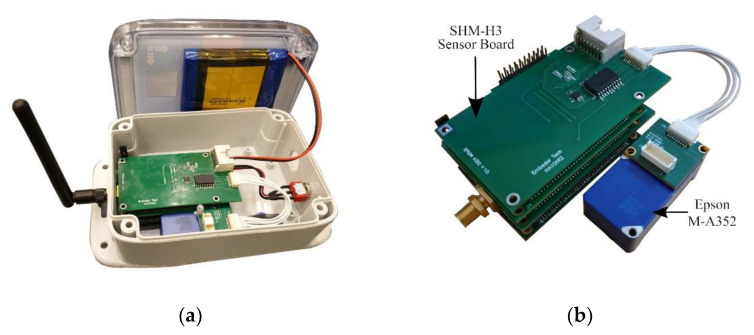
Xnode: (**a**) Xnode and enclosure with M-A352 accelerometer (**b**) Designed sensor board for M-A352.

The Xnode’s underlying hardware comprises three printed circuit boards (PCBs), which include a processor board, a sensor board, and a radio/power board. The processor clocks up to 204 MHz using an NXP LPC4357 microprocessor based on a dual-core ARM Cortex M4F/M0 architecture. The processor board also features a high-precision 12 MHz clock crystal with 30 ppm accuracy. The modular design of Xnode gives the flexibility to add-on external sensors with custom-built boards by stacking them onto the core, much like the shields used with Arduinos. Yet, the Xnode comes in an IP67 environmental rated hardened enclosure (see Figure 1a) for high-fidelity distributed sensing even in harsh environments while supporting solar-powered energy harvesting.

The Xnode software framework realizes preemptive multitasking—time scheduling of multiple tasks based on different priorities—using the real-time open-source operating system, FreeRTOS [36]. The middleware services are built upon the open-source Illinois SHM Services Toolsuite framework [15] and make use of an application specifically designed for distributed sensing demands in SHM, RemoteSensing. The three core tasks defined in the application include Sensing Task, Radio Task, and Application Task. They help facilitate reliable communication between a gateway and multiple sensors nodes in a network and synchronized data acquisition at high-sampling rates under low-power consumption constraints. A detailed overview of the core Xnode hardware and software framework can be found by referring to Fu et al. [29].

### 2.2. Integration of Digital Accelerometer and Xnode

Long-term condition assessment of civil infrastructures such as long-span bridges, dams, and underground/water tunnel networks based on the low-amplitude ambient vibration observed during periodic monitoring of these structures necessitates the need for high-sensitivity wireless accelerometers with µg resolution. Li et al. [4] highlight three design challenges in the development of a wireless high-sensitivity accelerometer which can be summarized as: (1) finding a suitable high resolution accelerometer to meet SHM application demands; (2) designing a low-noise PCB board; (3) developing a reliable device driver to support high-fidelity sensing. The authors identify a Quartz-MEMS (QMEMS) based high-precision digital output accelerometer, Epson M-A351, which has a superior low-noise level performance as an attractive option. However, in addition to the challenges mentioned above, moving towards digital output sensors for SHM brings forth a unique set of challenges for synchronized sensing in WSSNs. Digital accelerometers used for data acquisition often come with its own internal processor and clock crystal, thereby, adding in additional layers of uncertainties to consider for synchronized data acquisition between multiple such sensors. These challenges are discussed in detail and addressed in a following section (see Section 3).

In this study, a new-generation high-sensitivity digital accelerometer, the Epson M-A352, is integrated with the Xnode by building upon the research conducted by Li et al. [4] using its precursor version, M-A351. The notable features of M-A352 and a comparison with M-A351 is presented in Table 2. The M-A352 output has a resolution of 0.06 μg/LSB (average) with a wider dynamic range of ±15 g while supporting a frequency bandwidth up to 400 Hz. Moreover, it has a much lower startup time of 900 ms from the power-off state and a mere 16 ms from sleep mode, which allows it to better capture sudden events during monitoring. Typically, high-sensitivity MEMS-based accelerometers tend to be extremely fragile devices. Besides, it is common to observe mishaps and poor handling of sensors by engineers during field-deployment due to unforeseeable working conditions at the site, which makes it difficult to realize full-scale monitoring using these sensors. However, the improved shock resistance capability of the M-A352 up to 1200 g [37] makes it a more practical candidate for deployment in harsh environments for SHM.

#### 2.2.1. Hardware Support

The newly designed PCB board, SHM-H3 (see Figure 1b), to support the M-A352 accelerometer with the Xnode retains much of the partial-power down circuitry developed by Li et al. [4]; the CMOS buffer (SN74LVC, Texas Instruments) used allows a stable and low-jitter interface with the sensor. The sensor is securely mounted to the base of the Xnode enclosure (see Figure 1a), and a 9-pin wired connection establishes the link with the SHM-H3 board. In a continuous monitoring study of historic masonry towers using WSSs, Barsocchi et al. [39] highlight the need for having a well-designed mechanical casing and sensor mount to measure low-amplitude vibration. We carried out experimental tests to ensure that the sensor-casing interaction had minimal distortion to the measured response over a wide frequency bandwidth.

#### 2.2.2. Driver Support

The M-A352 is connected to the host platform using a UART serial interface for communication. The UART protocol runs at a baud rate of 460.8 Kbps using 8-bits for the data bit and 1-bit for the stop bit. Maintaining signal integrity in the communication channel by minimizing timing errors is crucial to avoid unexpected sensor behavior, especially during sensing at high data rates to avoid spikes due to corrupt packets. Supporting long-duration and high-data-rate acquisition on sensor nodes with limited resources is a nontrivial task. The challenges faced in this task can be broadly classified into three: (a) memory management, (b) reliable interrupts, and (c) low latency saving. Xnode has a volatile memory capacity of 32 MB RAM and for permanent storage supports 128 MB NAND flash along with 4 GB SD card storage. The high-resolution acceleration output by the M-A352 has a 32-bit (4 byte) data format, thereby saving each sample consisting of 3-axis data requires 12 bytes of memory. Accordingly, for a continuous ambient-vibration monitoring of sensing duration, say 30 min at a 1000 Hz sampling rate would require 21.6 MB. However, contiguous memory allocation of such a large size in dynamically allocated RAM may not be possible as the volatile memory is shared by other tasks running in parallel on the Xnode.

**Figure 2:**
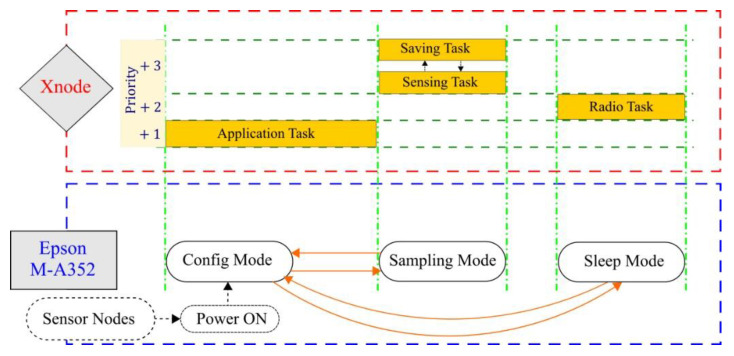
Xnode task handlers and corresponding mode of operation in M-A352.

Moreover, inefficient use of NAND flash can cause the sampling frequency of data acquired to be sporadically disrupted due to unexpected delays caused by flushing cache memory. Thereby, during sensing within a sampling interval of 1000 µs, we write data periodically to the NAND flash memory when the buffer size reaches the size of a NAND page, which takes about 760 µs, leaving enough slack time to read the next sample from the sensor. The flow between Sensing Task and Saving Task is controlled using interrupts and semaphores to ensure that no data sample is missed. The Xnode task handlers supporting the three modes of operation corresponding to M-A352, namely, Configuration mode, Sampling mode, and Sleep mode, are illustrated in Figure 2.

The other significant enhancements to the developed software framework over the previous version on the Xnode include support for time-synchronized sensing (see Section 3.3), power-saving sleep mode, and user-defined programmable Finite Impulse Response (FIR) filters. The developed driver allows the gateway node to remotely command the M-A352 within the sensor nodes to go into a power-saving sleep mode with an average current draw of only 1.3 mA. This allows fast wake-up to sudden events, either using the in-built threshold detection feature of M-A352 or an external trigger pulse from the Xnode. Besides, the driver provides control to the user to change the FIR filter used by M-A352 based on the application requirement by sending commands over the gateway node. For instance, in sudden event monitoring such as earthquakes and bridge impacts, the initial data in the first few milliseconds of the event is crucial. Thereby, in such a use case scenario, we can minimize the transient delay by using a lower number of taps for the FIR filter. In addition, in the case of long-term monitoring of structures such as bridges with a known lower-frequency bandwidth of interest, we can utilize a custom-designed filter with a faster roll-off that can run at lower sampling rates, which would directly translate into more power and memory savings in WSSs. Altogether, remote control over a user-customizable FIR filter within digital output sensors facilitates a more efficient usage of WSSs for a broader range of tasks.

## 3. Time Synchronization with Digital Accelerometers

Time synchronization is critical to the effective operation of WSS device networks for applications such as monitoring and real-time control systems. Time-critical SHM applications such as damage identification demand high synchronization accuracy levels while being constrained by battery life and computing resources available on the WSS. Digital sensors used for data acquisition on the WSSs often come with their own internal processor and clock crystal, thereby adding in an additional layer to consider for synchronized data acquisition between multiple such sensors on WSS devices. In this section, we provide a brief overview of time synchronization strategies in WSSNs for SHM; additional challenges encountered in data synchronization by incorporating digital accelerometers are discussed in detail, and an algorithm is proposed to characterize these uncertainties in WSSs.

### 3.1. Challenges with Digital Accelerometers

Achieving synchronized sensing among sensor nodes in a WSSN is a nontrivial task; the inclusion of digital accelerometers to the network introduces a unique set of challenges to achieve synchronized data acquisition. Indeed, there are many benefits of using digital accelerometers, as highlighted in Section 2.2. Though, it also brings forth additional uncertainties in delays and internal latencies associated with the internal processor, which makes it more challenging to obtain time aligned data samples among sensors. Errors from data synchronization between sensors can be of two types: random and deterministic errors; random errors can be described as delays that are not repeatable in nature for the same setup and can include clock drift, interrupts, sensing command beacon arrival, internal processor latencies, etc. The primary sources of deterministic errors include filter group delays, clock offset, and clock frequency deviation. Digital accelerometers make use of an internal ADC to convert the measured physical quantities to digital signals for further processing. The latency which relates to the ADC is the time taken for an analog sample to be clocked in, processed, and output digitally to the internal processor [40,41]. Besides, there are delays associated with the internal processor for the digital sample to undergo signal conditioning and calibration before an interrupt signal asserts a Data Ready (DRDY) pin and the output gets pushed to a serial interface for host access.

**Figure 3:**
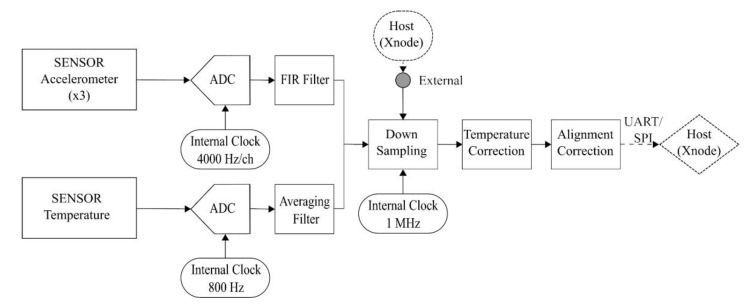
Functional block diagram for Epson M-A352 [37].

**Figure 4:**
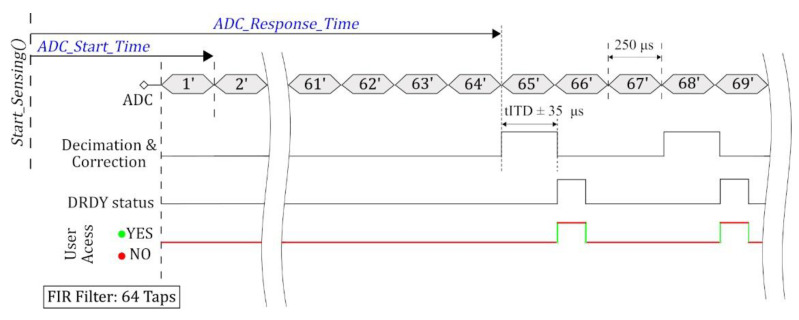
Representative timing diagram for Epson M-A352.

Typically, in low-power digital accelerometers, only limited access to the internal ADC is provided for the host, which prevents precise timestamping of the analog sample points [37,38,42]. An oversampling approach is used with ADCs for achieving a good performance to cost ratio in which the input signal gets sampled at a much higher rate (typically by a factor of four) than the Nyquist frequency to improve the resolution and signal to noise ratio (SNR) [43,44]. Manufacturers generally do not provide direct bus access to the high-frequency ADC output as it often results in more power consumption and increased internal processor demands, especially in the case of sensors which are geared towards low-power consumption requirements in their design. So, the ADC output gets downsampled as per user requirement before timestamping access is provided to the host. The downsampling can be controlled either using an internal crystal oscillator (XO) or an external input-signal from the host. The subsampling approach using the internal XO can be referred to as the standard mode of operation with digital sensors, in which the XO generates electrical signals at precise frequencies controlled by the internal processor of the digital sensor without the need for any host interference. The other alternative of external trigger (EXT) controlled downsampling utilizes host-generated trigger pulses at user-defined frequencies. It provides more flexibility and control to the host over timestamping of the generated pulses, although it comes at the expense of additional workload for generating these interrupts externally. The functional block diagram of a digital accelerometer, Epson M-A352, with host interaction is shown in Figure 3. It illustrates the workflow of acquiring an analog signal and sending the digitized sample to the host. The delay from the time a sensing command is sent by the host until the ADC finishes clocking in the first analog sample; this can be referred to as *ADC_Start_Time*, depicted in Figure 4. The uncertainty pertaining to *ADC_Start_Time* is mainly dependent on the overhead latencies from internal processing within the digital sensor. The digitized samples pass through a FIR filter adding on a deterministic filter transient response delay (including group delay) before the signal undergoes downsampling and correction.

**Figure 5:**
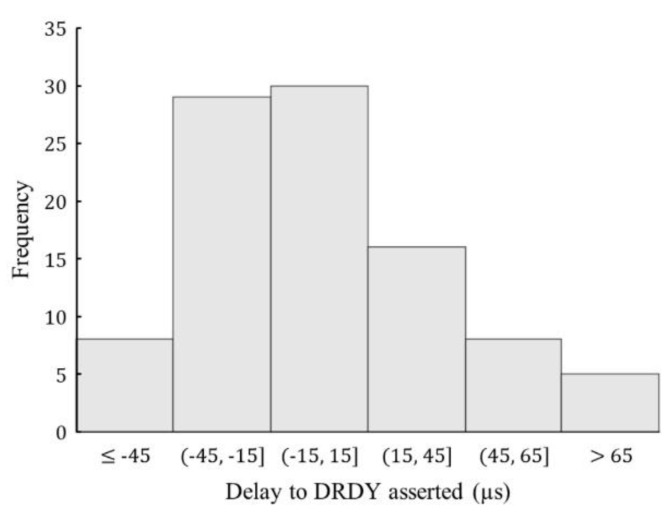
Histogram plot of mean-normalized delay in Data Ready (DRDY) for 100 runs.

Figure 4 depicts a representative timing diagram (with 64 Taps FIR Filter) for Epson M-A352 and user access to timestamps of a sample being processed by the sensor in different stages. Note that the first ADC sample available to the user is only after the transient response of the FIR filter, the delay to this sample from the time the *Start_Sensing()* command is initiated by the host is referred to as *ADC_Response_Time*. The host does not have access to the accurate timestamp when the signal gets downsampled; it can be registered only when the DRDY pin gets asserted, after downsampling and correction, which has a nondeterministic delay with a max value of 430 µs [37] in normal-mode of operation. Moreover, an uncertainty ranging up to ±35 µs is observed in this delay while using the 64 Taps filter setting, as illustrated by the histogram plot (Figure 5) of 100 runs showing the mean normalized delay from *Start_Sensing()* to the first DRDY getting asserted. In addition, the upper bound of this uncertainty is observed to be dependent on the sensor startup settings and presets for multiple runs, reaching up to ±250 µs. Although, this range was reduced with additional software-reset commands to the sensor reducing the overhead latencies. Even though an error in the order 10′s of microseconds may seem small for a particular sensor, it can still be considerable for some of the SHM applications, as discussed in Section 1.

The data synchronization technique based on the resampling approach, discussed in Section 1, does not compensate for this uncertainty in *ADC_Start_Time* as it relies on the assumption that the correct timestamp corresponding to a sample is accessible, which is not necessarily the case with digital output sensors. Studies considering this nonzero random uncertainty in delay within WSSNs have been lacking. If left uncompensated, it can directly add to the overall time synchronization error in the network, especially in the case of WSSNs employing different types of external sensors in the same network.

### 3.2. Adaptive Algorithm for ADC Startup Delay Estimation in WSSs

**Figure 6:**
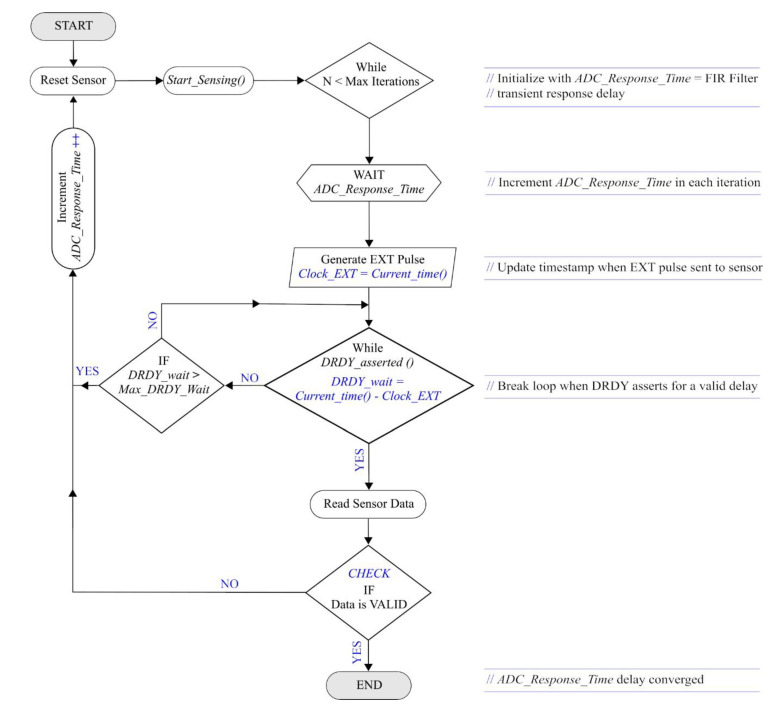
Analog to Digital Converter (ADC) startup delay estimation algorithm.

We propose an iterative algorithm, illustrated in Figure 6, to estimate the *ADC_Start_Time* delay in digital output sensors for accurate timestamping of samples. The approach can be implemented on WSSs without the need for any dedicated evaluation setup and direct access to the internal ADC.

**Figure 7:**
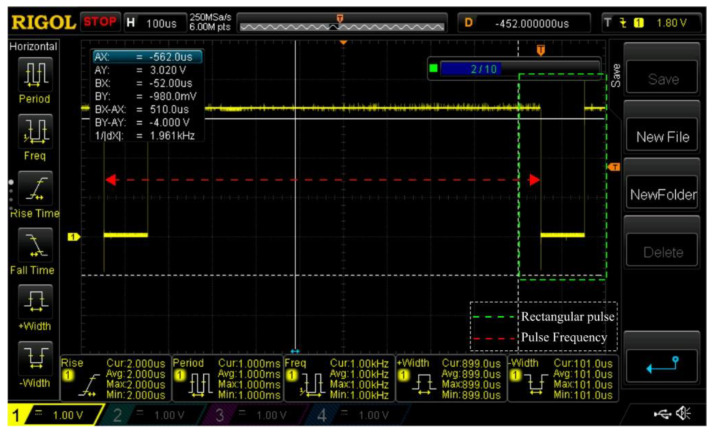
Oscilloscope view of EXT pulses generated at 1000 Hz by the Xnode.

In the algorithm, at the start, we initialize the *ADC_Response_Time* with a minimum value corresponding to the transient response delay of the FIR. This wait delay is implemented with a 12 MHz XO tick precision on the Xnode, then the EXT pulse is sent out to the sensor, and we poll for the DRDY value. Figure 7 depicts the oscilloscope view of a series of EXT pulses of 1 µs width (highlighted in green) generated at 1000 Hz by the Xnode. At this stage, we wait either until DRDY is asserted or if it exceeds the maximum wait time typically specified in the datasheet for the DRDY interrupt (740 µs for M-A352 [37]). If the DRDY gets asserted the sensor output is read, a valid value indicates the convergence of *ADC_Response_Time*. Otherwise, we increment the *ADC_Response_Time* by an amount less than or equal to the jitter tolerance of the EXT pin (5 µs for M-A352 [37]) and iterate again after resetting the sensor until we obtain the first valid ADC sample indicative of convergence. The *ADC_Start_Time* can be determined using Equation (1) shown below by accounting for the transient response delay of the FIR filter.

(1) ADC_Start_Time=ADC_Response_Time−(N−1)×Sampling_Interval

where *N* is the number of FIR filter taps, and *Sampling_Interval* is the period of the internal XO sampling the ADC, which is 250 µs in our case. 

**Figure 8:**
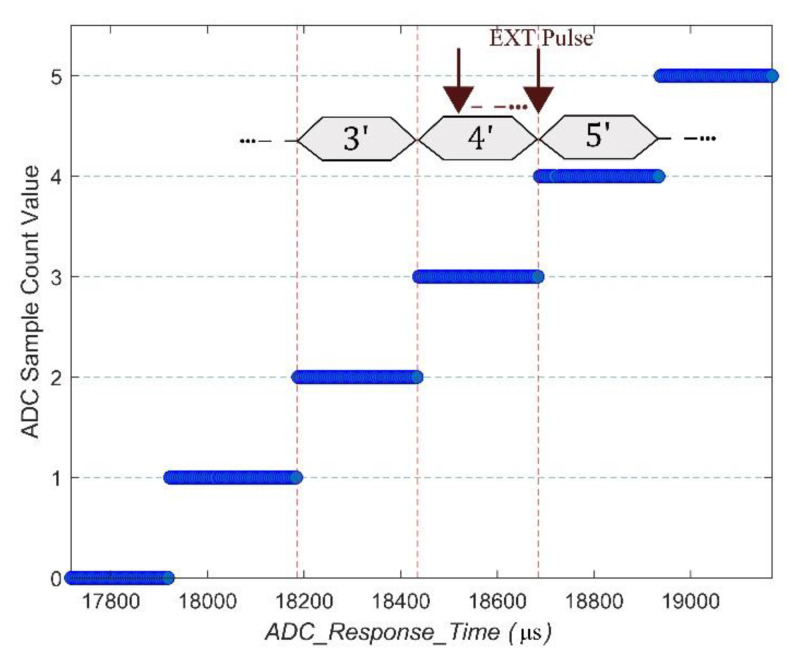
Timing diagram of ADC Startup delay evaluation with Epson M-A352.

The behavior of the algorithm is demonstrated through an evaluation carried out on the Epson M-A352 at room-temperature working conditions. An illustration of the ADC sample count value and corresponding timestamp obtained by extending the algorithm for a test with 1000 Hz sampling rate and sensing parameters set to a FIR Kaiser filter (64 taps) is depicted in Figure 8. The ADC sample count value refers to a counter value register provided on the M-A352 [37], which gets incremented based on the sampling completion timing of the internal ADC. It has been used to simply visualize the current ADC sample number, which gets downsampled by the EXT pulse, and the corresponding *ADC_Response_Time*. The internal ADC gets sampled at a fixed interval of 250 µs, downsampling the signal from 4000 Hz to 1000 Hz can be seen as sampling every four consecutive ADC sample output. When using the EXT pulse to downsample, the challenge concerning the first sample is that the accurate delay in timing required before triggering the pulse from the point a *Start_Sensing()* command is invoked remains unknown. Moreover, we need to precisely trigger the pulse at the transition region between two ADC sample outputs, as highlighted in Figure 8. Note that for clarity, only the ADC sample count values up to 5 are shown in the above. 

The M-A352 manufacturer (Seiko Epson Corporation, San Jose, CA, USA) supplied *ADC_Start_Time* nominal timing data characterized at room temperature as requested by the authors for validating our algorithm testing. The *ADC_Start_Time* is evaluated using the proposed algorithm for different FIR filter taps setting, and the error in reference to the values reported by the manufacturer is presented in Table 3. We observe that the maximum error is under five µs, demonstrating the accuracy of the proposed algorithm.

For applications such as long-term periodic monitoring in SHM, where a quick-startup time of a sensor node is not critical and the focus is more on high-precision synchronized sensing, the proposed strategy can be used to estimate the errors just before the startup of sensing cycle. The convergence time of the method is dependent upon the number of FIR filter taps setting and the initial estimate used while starting the iteration. Startup time-constrained sudden-event monitoring applications can utilize offline-executed and saved offset estimates with a standard deviation for compensation. A reset command is used at the beginning of the algorithm to minimize the jitter in the estimation, while it is typically the most time-consuming block of the algorithm. In power consumption constrained scenarios for faster online execution, the algorithm can skip the reset command block to give an approximate estimate, under 500 ms in the case of the above evaluation. The average current draw of Xnode with the M-A352 is 180 mA, as compared to some of the other standard WSSN platforms for SHM, such as the Martlet (Kane et al. [20]) and Imote2 (Adler et al. [45]) with 190 mA and 168 mA respectively. A study by Luczak et al. [46] on the aging effects of MEMS accelerometers indicates that the changes to typical offsets and bias in these devices can be significant due to aging effects, especially under harsh operating conditions. The proposed method is expected to adapt to the actual working conditions of the sensor, such as operating temperature to give a better estimation rather than a nominal value, as it does not use any preset fixed delay values for the ADC delay characterization. Although, the precision would be dependent upon the jitter range of EXT pin. An end-user is generally interested in the *ADC_Response_Time* delay of a sensor for their application, rather than getting into the intricacies of the ADC specifications or the ADC—Internal processor interaction details within the sensor which are typically considered proprietary information by the manufacturers. The proposed approach only utilizes commonly provided features in digital accelerometers, namely, EXT and DRDY pins, thereby, can be generalized to most of the digital accelerometers on the market for ADC delay characterization for time-critical applications using WSSs.

### 3.3. Synchronized Sensing Strategy Implementation

Recent work by Fu [22] on time synchronization using the standard Xnode sensor board (analog accelerometer) shows promising results with an average error of 10 µs. The authors proposed an efficient clock synchronization strategy using only two rounds of point synchronization (before and after sensing) and compensating for the timestamping errors. The nonlinear clock drift is compensated by using the secant slope of the clock drift curve. In this study, we implement the above approach for clock synchronization in the network.

**Figure 9:**
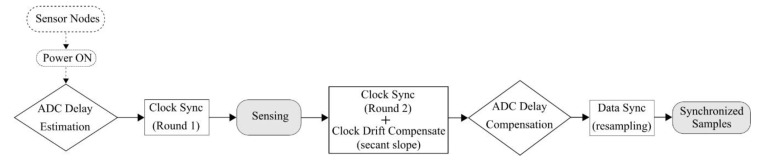
Flowchart of synchronized sensing framework.

The data synchronization strategy using the resampling-based approach proposed by Nagayama and Spencer [13] allows us to ensure the sensing command is executed at nearly the same time on each node using global timestamps. Extensive testing by Fu [22] showed that the aforementioned challenges (2) and (3) identified in Section 1 are minimal with the Xnode, relating it to the high-precision and stability of its clock crystal. However, incorporating external digital output sensors in WSSs increases the uncertainty of the delay in the actual start of sensing in each node. Specifically, it is required to characterize the delays in the startup of the internal ADC of digital output sensors. Thereby, the above approach alone will not necessarily achieve a synchronized start of sensing when external digital output sensors are involved in a WSSN. In summary, the challenges to account for digital output sensors in WSSs include: (a) same as those in conventical sensors; (b) unique startup delay uncertainty. We address (a) by incorporating Fu’s [22] method for clock synchronization and the approach by Nagayama and Spencer [13] for data synchronization. Uncertainties in delay introduced by (b) are characterized using the algorithm proposed in Section 3.2 of this paper. The synchronized sensing framework developed in this study by incorporating external digital output sensors with the Xnode is illustrated using the flowchart shown in Figure 9.

## 4. Experimental Validation

The low-amplitude vibration measurement capabilities and time synchronization performance of the developed wireless accelerometer are evaluated through experimental testing in this section. Ambient vibration tests were conducted to assess its performance across the wide-frequency bandwidth available. We carried out experiments with multiple sensors secured to a Load and Boundary Condition Box (LBCB) and subject to band-limited white noise (BLWN) to evaluate the level of time synchronization achieved using the synchronized sensing framework proposed in this paper.

### 4.1. Ambient Vibration Tests

#### 4.1.1. Low Amplitude Vibration Laboratory Test

**Figure 10:**
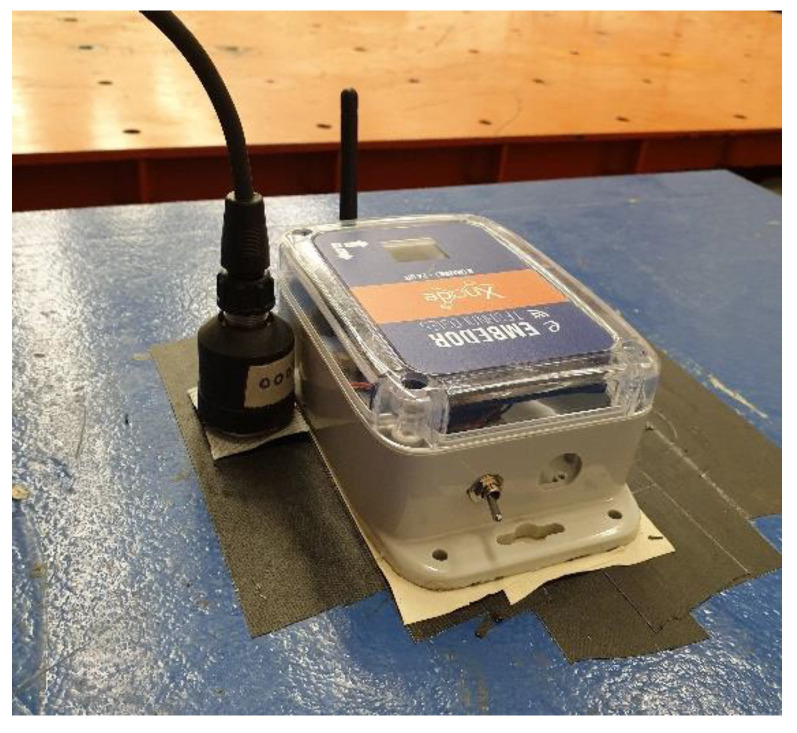
Ambient vibration test setup in the lab.

The performance of the wireless digital accelerometer for low-amplitude vibration was evaluated using a test setup (shown in Figure 10) in the basement of Newmark Civil Engineering Laboratory, the University of Illinois at Urbana-Champaign (UIUC). The reference measurement was taken using a wired high-sensitivity piezoelectric accelerometer, PCB393B12 (PCB Piezotronics, Inc., Depew, NY, USA), which features a low-noise level average of 8 µg/RMS over 0.15–1000 Hz. The test was carried out at late night to minimize the environmental noise due to machinery in the building and departmental activities by researchers. The Xnode with Epson M-A352 and the reference wired sensor were affixed to the top of a relatively flat concrete block using the same material (duct-tape) to collect 120 s of ambient vibration data, such that the measured input is almost identical. The sampling rates used by the wireless accelerometer and the wired sensor were 1000 Hz and 1024 Hz, respectively. The internal filter settings of A352 were set to a default FIR Kaiser window-based filter of order 512 and a cut-off frequency of 460 Hz. For analysis, the acquired data is passed through an 8-pole elliptic low-pass filter with filter cut-off at 400 Hz. 

**Figure 11:**
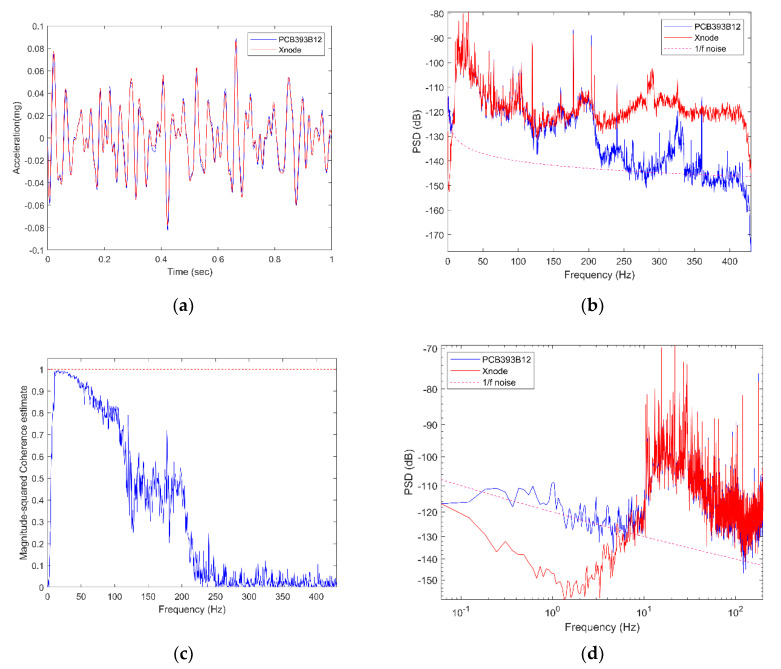
Basement lab ambient vibration test results: (**a**) zoomed-in view of time-history plots for the cut-off frequency of 50 Hz (**b**) Squared-Coherence estimate between the reference and wireless accelerometer (**c**) PSD plot of acceleration records (**d**) PSD comparison in log-log scale.

Figure 11a shows a zoomed-in view of the time-history response with a filter cut-off set at 50 Hz for clarity, illustrating the excellent correlation obtained between the signals with an amplitude of 0.08 mg. Figure 11b,c shows the Power Spectral Density (PSD) of acceleration records and Magnitude-square of coherence between the signals, respectively. In the higher-frequency range up to 115 Hz, there is good agreement between the PSD records with the coherence estimate value above 0.7. For this test setup, the amplitude of signal content in the very high-frequency range above 115 Hz was minimal such that PSD obtained by the two sensors appears to diverge, merely because the wired PCB393B12 accelerometer has a better noise floor than the wireless accelerometer in this high-frequency range. Theoretically, the PSD plot of acceleration approaches zero (negative infinity in log scale) at zero frequency. In the very-low-frequency range of 0–10 Hz, we observe that the wireless digital accelerometer has much lower noise than the wired reference sensor and can better capture the trend near DC-response (see Figure 11d). In addition, we can observe that the graphs follow the 1/f or “pink” noise limits observed in electronics when close to the DC range [47].

#### 4.1.2. Ultra-Low Amplitude Vibration Field Test

**Figure 12:**
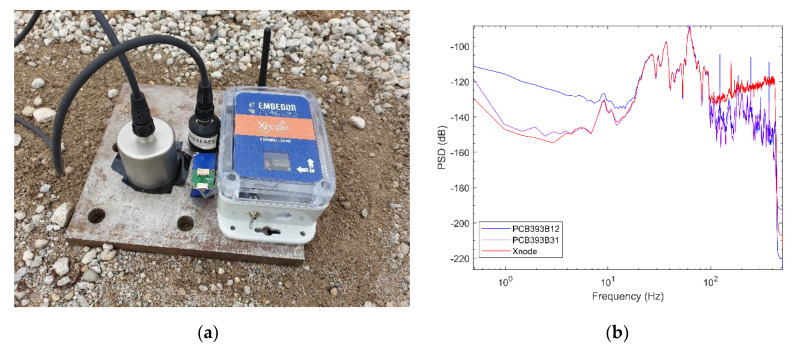
Field ambient vibration test results: (**a**) test setup using Xnode—M-A352 with two reference sensors (**b**) PSD plot of acceleration records.

For further assessment of the performance close to the noise-floor limits of the wireless accelerometer, a field test was also conducted in which the amplitude of vibration of signal content within 50 Hz was observed to be in the order of 2 µg. The test was performed during the early morning hours of Thanksgiving Day on a countryside road near Mahomet, Illinois, away from busy routes with minimal traffic-induced ground vibration. In the test setup (see Figure 12a), a higher-sensitivity reference sensor, PCB393B31, featuring noise levels of 1 µg/RMS over 0.1–200 Hz, was used in addition to the reference sensor used for the above laboratory study. The standard noise floor mode of the M-A352 with sensing parameters of the setup same as those used for the laboratory test was used.

Figure 12b shows a good correlation between M-A352 and the wired PCB393B31 in the range of 5~100 Hz. Notably, the wireless accelerometer has a better performance than even the wired PCB393B31 reference sensor in the very-low-frequency range of 0~5 Hz. Although, above 100 Hz, it is observed that the amplitude level of signal content is smaller than that which could be measured accurately with M-A352 given its noise-floor limits in that range. Subsequently, a linear trend is observed above 100 Hz for M-A352 in the PSD plot in accordance with the noise density characteristic in the datasheet [37].

### 4.2. Synchronization Tests

**Figure 13:**
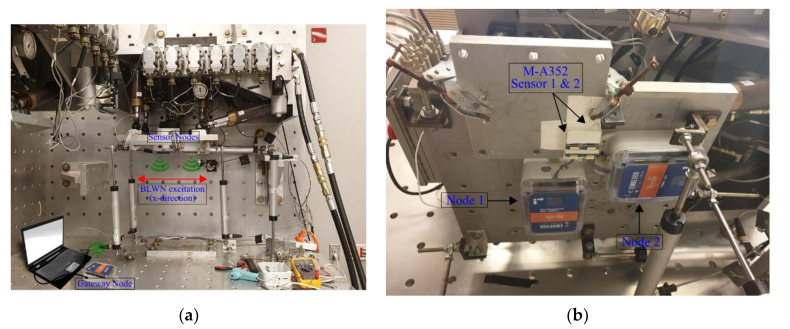
Test-setup for time synchronization error evaluation: (**a**) Load and Boundary Condition Box (LBCB) with test-setup (**b**) Two Xnode sensor nodes with M-A352 mounted to a base plate.

We evaluate the time synchronization performance of Xnode integrated with the high-sensitive digital accelerometer in this subsection. The experimental setup consisted of a gateway node and two sensor nodes with an Epson M-A352 high-sensitivity accelerometer on each. The sensor nodes were installed onto the 1/5th-scale Load and Boundary Condition Box (LBCB) in the Network for Earthquake Engineering Simulation (NEES) Studio, UIUC. We mounted the accelerometers securely to a base plate, shown in Figure 13b, such that a nearly identical input vibration is measured by both the sensors. The test setup is subjected to band-limited white noise (BLWN) excitation input with 0–20 Hz bandwidth using the LBCB in the x-direction (see Figure 13a). A perfectly synchronized measurement of a BLWN signal by the sensors would result in a zero-phase difference, thereby, a nonzero slope of the phase angle obtained from the cross power spectral density (CPSD) between the pair of sensors is indicative of time synchronization error ($TS_{error}$), which is estimated in µs using Equation (2).

(2) $$TS_{error}=\frac{\theta_{phase}}{2\pi }\times 10^{6}$$

where $\theta_{phase}$ is the slope of the straight-line fit using linear regression on the phase angle curve.

**Figure 14:**
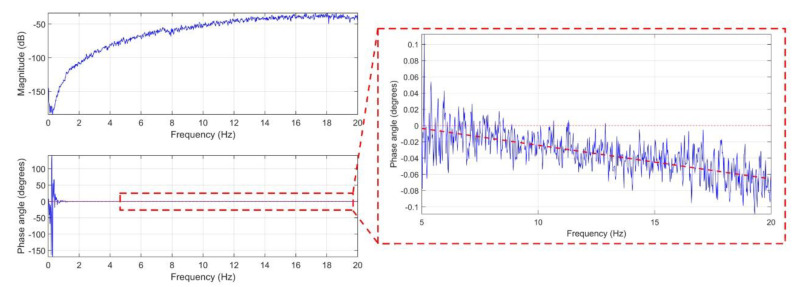
Cross power spectral density (CPSD) and phase diagram for a 15 min duration LBCB test.

The recorded signals from sensor nodes 1 and 2 (see Figure 13b) were corrected for corresponding offsets in startup sensing time and drift with respect to global timestamps using the synchronization approach proposed in Section 3.3. For illustration, the CPSD and phase diagram between the synchronized signals of Node 1 and Node 2 obtained for a 15 min duration test with the LBCB is shown in Figure 14. Note that for the above tests, the input signal to the LBCB was a displacement controlled BLWN signal of bandwidth 0–20 Hz. However, our sensors measure the acceleration response of the LBCB, which in the frequency domain varies as the square of frequency times the Laplace transform of BLWN displacement. Thereby, a pure BLWN with a perfectly flat magnitude is not obtained in the low-frequency range, as seen from the CPSD magnitude shown in Figure 14. Although, for our evaluation purposes the signal content from 5–20 Hz does give the expected linear trend which correlates to $TS_{error}$ between the sensors. Accordingly, we fit the straight line using linear regression on the phase diagram from 5 Hz onwards (highlighted in red in Figure 14), and the slope is translated to synchronization error using Equation (2). We evaluated the $TS_{error}$ for different sensing durations (1 min, 15 min, and 30 min) to simulate short and long duration sensing. In each case, the results over three trials, along with the average synchronization errors with and without adopting the proposed compensation strategy, are reported in Table 4. In the above tests, the sensor parameters were set to use a FIR filter of 64 taps with a 1000 Hz sampling rate.

It can be seen that the average synchronization error for long-duration sensing with the proposed strategy is less than 15 µs. It may be noted that this is a significant improvement in data synchronization error over the previous works (Table 1) using digital accelerometers with WSSs. The achieved precision can be attributed to the precise handling of uncertainties with the proposed iterative algorithm and the robustness of the proposed time-synchronization strategy implemented on the Xnode.

## 5. Conclusions

This paper proposes a synchronized sensing framework for wireless smart sensors (WSSs) employing digital accelerometers. The unique set of challenges and additional uncertainties introduced by digital accelerometers in achieving synchronized sensing are identified and addressed. We present an adaptive iterative algorithm to characterize the startup delays in external digital accelerometers with limited ADC access using WSSs. A high-sensitivity digital accelerometer is integrated with the Xnode WSS platform for synchronized long-term periodic monitoring. The wide-bandwidth, low-amplitude ambient vibration sensing capabilities of the node are validated through laboratory and field tests. The maximum average data synchronization error of the developed WSSN is shown to be under 15 µs with the experimental validation carried out using an LBCB subject to BLWN. The proposed synchronization approach can be more broadly extended to other external digital output inertial sensors and sensor platforms, thus improving reliable data collection in full-scale long-term monitoring deployments for more accurate condition assessment. 

## Figures and Tables

**Table 1:** Summary of time synchronization error of wireless smart sensors (WSSs) using digital accelerometers.

Study	Year	Error	
		Clock Synchronization ^1^	Data Synchronization
Bocca et al. [27]	2011	~10 µs	-
Valenti et al. [9]	2018	-	~6 ms
Narayanan [25]	2019	-	~50 µs ^2^
Navabian and Beskhyroun [28]	2020	-	2 ms

^1^ small clock sync error does not necessarily imply a small data sync error. ^2^ with access to the internal ADC timestamps.

**Table 2:** Comparison between M-A352 and M-A351 accelerometers [37,38].

Feature	M-A351	M-A352
Custom Programmable FIR filters	No	Yes
Sleep mode (1.1 mA usage)	No	Yes
Max sampling rate	500 Hz	1000 Hz
Frequency bandwidth	100 Hz	400 Hz
Shock resistance	300 g	1200 g
Power-on to Start-up time	2 s	900 ms
Output dynamic range	± 5 g	± 15 g
Supply current (Avg.)	20 mA	13.2 mA
Noise (Avg.)	0.5 $\mu g/\sqrt{\mathrm{Hz}}$	0.2 $\mu g/\sqrt{\mathrm{Hz}}$

**Table 3:** ADC startup delay estimation for different filter taps setting.

FIR Filter Taps	*ADC_Start_Time* (µs)	Error (µs)
64	2937.35	2.35
128	3202.33	1.33
512	4787.31	4.31

**Table 4:** Time-synchronization results from LBCB tests.

Sensing Duration	Synchronization Error (µs)				
	Trial 1	Trial 2	Trial 3	Average	Average without Compensation ^1^
1 min	6.12	9.76	7.98	7.95	80.44
15 min	12.47	13.47	11.52	12.49	111.3
30 min	17.26	11.23	14.32	14.27	135.87

^1^ Average sync-error without ADC delay and drift compensation.
